# Adaptive designs for trials aiming to optimise implementation strategies and the effect of an additional interim analysis: a simulation study

**DOI:** 10.1186/s12874-025-02730-y

**Published:** 2025-11-29

**Authors:** Erin Nolan, Daniel Barker, Elizabeth Holliday, Joshua Dizon, Alix Hall, Christopher Oldmeadow

**Affiliations:** 1 School of Medicine and Public Health, The University of Newcastle, Callaghan, NSW Australia; 2 Hunter Medical Research Institute, New Lambton Heights, NSW Australia; 3 National Centre of Implementation Science, The University of Newcastle, Wallsend, NSW Australia; 4 Hunter New England Population Health, Hunter New England Area Health Service, Wallsend, NSW Australia

**Keywords:** Implementation, Trial design, Bayesian, Adaptive designs, Optimisation

## Abstract

Trials that aim to optimise the implementation of an intervention are often complex, requiring multiple, combined strategies and requiring uptake on multiple levels. Previous implementation trials have optimised implementation strategies using multi-arm cluster randomised control trials (cRCTs) but can be inefficient and waste resources. Adaptive designs may potentially improve efficiency of these trials, but under what design features and trial properties is unknown. A simulation study was performed to assess under what conditions, if any, one or two interim adaptive designs offer increased efficiency compared to a ‘fixed trial’ approach for implementation cRCTs. A four-arm cRCT was simulated with varied trial properties, with a fixed design or an adaptive design (varying by number of interim analyses and timing of interim analyses) and modelled using Bayesian hierarchical models. The adaptive design allowed for stopping early for futility and dropping an arm for futility. The power, type 1 error rate, and adaptive design decisions were compared between the designs across trial properties. Both one and two interim adaptive designs offered power gains and a lower type 1 error rate compared to the fixed designs across most trial properties. A high intra-class correlation (ICC) of 0.2 led to adaptive trials dropping effective arms or incorrectly stopping for futility more frequently, with the incorrect decisions being compounded when two interim analyses were used. The rate of these incorrect decisions was reduced when the first interim analysis in the two interim designs was delayed. Adaptive designs can offer improved efficiency, more power, and reduce resource wastage for trials compared to fixed designs.

**Supplementary Information:**

The online version contains supplementary material available at 10.1186/s12874-025-02730-y.

## Background

Implementation trials aim to translate evidence-based best practice into standard care [1, 2]. The process of implementing an intervention is complex, often requiring multiple, combined, strategies to maximise effectiveness. Despite best efforts, the efficacy or effectiveness of implementation strategies often attenuate over time due to limited resources, such as staff time or funding [3]. The process of optimisation, where an intervention or its implementation is iteratively improved, guided by data and within real-world constraints, may more efficiently find which combination of strategies maximise effectiveness, while limiting attenuation over time by considering real world constraints [4].

Adding to the challenges of maintaining effectiveness of the intervention, implementation trials can have complex designs, often requiring uptake of strategies at multiple levels (e.g. students, teachers, schools) [1]. Current designs used to optimise implementation strategies are often inefficient and carry significant limitations. For example, some previous implementation trials have optimised strategies over multiple, sequential, two-arm cluster randomised control trials (cRCTs), with each trial evaluating one component of an implementation strategy at a time, with the results informing adaptations made to improve the implementation strategy that are then tested in the subsequent trial [5]. Such designs can be inefficient, compared to designs that assess multiple strategies simultaneously [6–10]. Other designs such as multi-arm cluster and individually randomised control trials (RCTs) – which can assess multiple components within a single trial – have been used to optimise implementation strategies [11–13]. In multi-arm trials, the different components of the strategy can be treated as distinct arms. However, not all components are necessarily assessed, but instead a subset of components may be examined. These multi-arm designs allow evaluation of multiple components (albeit not their interactions) with potentially lower trial cost than multiple, sequential 2-arm RCTs, and lower cost than factorial designs if the cost per condition is high [14]. Factorial designs are mainly used to assess combinations of treatment components (including their interactions) and have also been recommended for optimisation [14]. Despite the potential efficiency gains multi-arm trials offer, they can still be costly, time consuming and risk being underpowered in practice, despite best efforts at recruitment [11, 12].

Cluster randomised trials, unlike individually randomised trials, randomise whole clusters to a treatment arm, often to minimise the risk of treatment contamination [15] or because the intervention by its very nature can only be delivered at the cluster level [6, 7]. A feature of cluster randomised trials is correlation of response values among participants within a cluster; violating assumptions of independence. The correlation of response values between participants within a cluster is quantified by the intra-class correlation coefficient (ICC). This correlation is inversely related to power, meaning as the ICC increases, power to estimate a given effect size for a fixed cluster size decreases. The historical challenges of sequential two-arm trials in optimising implementation strategies, along with the potential low power and unique design considerations of cluster randomisation, warrant the exploration of other designs to improve trial efficiency.

Compared to fixed designs, adaptive designs may offer increased trial efficiency, decreasing the time and sample size required to reach a conclusion about the optimisation of the intervention or implementation strategy [16, 17]. Unlike fixed designs, which don’t analyse trial data until the end of the trial, adaptive designs use accumulating data to make pre-specified decisions during the trial [18]. These decisions are made using the results of interim analyses and may include stopping the trial early for efficacy or futility, dropping or adding one or several treatment arms, or increasing the projected sample size. The number and timing of interim analyses must be specified prior to the start of the trial. Using interim analyses to make adaptive design decisions can affect the length, complexity, and outcomes of the trial. Therefore, the use of interim analyses needs to be accounted for in the trial design [18]. The decision on the number of interims can be particularly important, as the number of interims has been demonstrated to affect key performance measures such as power and the type 1 error rate. For example, additional interim analyses when stopping for futility can decrease the power and type 1 error rate [19, 20]. Considering the additional time and burden that an interim analysis can pose on a trial, it is important to consider the potential benefit, if any, of any candidate adaptive design.

While many previous trials have used frequentist methods to evaluate optimisation, the Bayesian framework has been used with success [16]. Bayesian models have been used to evaluate the optimisation of implementation strategies previously, and can offer more informative conclusions than frequentist methods [6]. Bayesian models take prior information and current trial data to create a posterior distribution of the treatment effects. These models can incorporate historical information, such as results from a pilot trial, into the distributions summarising prior information [21]. The Bayesian framework is useful for cRCTs as it can offer higher power and a lower type 1 error rate under some scenarios [22]. Type 1 error and power are often considered strictly frequentist characteristics, not applying to Bayesian analysis. However, even in a Bayesian framework, making design decisions based on the accumulated data in an interim analysis has the ability to impact the type 1 error rate and power [20]. Considering these typically frequentist operating characteristics when designing a Bayesian trial is both recommended [23] and common [18] in medical research.

The adaptive design of early stopping for futility and/or dropping an arm for futility, otherwise known as a multi-arm multi-stage (MAMS) design, has been widely explored [24, 25]. This design has been previously applied in clustered randomised trials [24], and within a Bayesian framework for individually randomised trials [26, 27]. Previous sequential two-arm cRCTs that optimised implementation strategies, such as the PACE trials [6, 7], illustrate a type of study that has the potential to benefit from the Bayesian MAMS design. The PACE trials, which optimised a complex implementation strategy over multiple trials, might have more efficiently optimised if they were conducted as a multi-arm trial that could stop early for futility or drop inefficient arms. However, the literature on the use of Bayesian MAMS designs for clustered trials is sparse. In addition, this design has not been proposed for use in trials aiming to optimise an implementation strategy. This paper contributes to the literature by exploring the use of Bayesian MAMS designs for a cRCT focused on optimising an implementation strategy.

The question arises as to how adaptive designs behave for cRCTs aiming to optimise implementation strategies, and whether additional interim analyses in adaptive designs provide any benefit to multi-arm cRCTs that aim to optimise implementation strategies. Running a simulation study can offer insights regarding this question. A simulation study emulates what may occur in a real trial when varying different designs and trial properties. From this, we can determine the potential impact these candidate designs may have on a variety of trials. One candidate adaptive design is explored in this study: a design with both early stopping for futility (i.e. stopping all arms) and arm dropping (i.e. stopping one arm). By dropping ineffective arms, the power to detect an effect in the remaining arms should increase. However, one risk of adaptive designs is that the interim analysis is performed too early and the design decision drops a truly effective arm. The timing of the interim analysis should be explored to examine whether the risks of multiple interim analyses can be reduced by delaying the first interim. By defining a discrete number of realistic thresholds for declaring futility and dropping an arm, simulation results can be displayed clearly. In practice however, thresholds should be set to ensure the type 1 error rate is maintained but not overcontrolled [28].

### Aims

1. Examine under what trial properties, if any, adaptive designs affect the efficiency and key performance indicators (power, type 1 error rate) in cRCTs seeking to optimise implementation strategies.
2. Examine under what trial properties, if any, the number of interim analyses in an adaptive design affects the efficiency and key performance indicators (power, type 1 error rate) in cRCTs seeking to optimise implementation strategies.

## Methods

The ADEMP (aims, data generating mechanisms, estimands, methods, performance measures) and Bayesian simulation study (BASIS) frameworks [29, 30] were used to guide the structure of the simulation study.

### Aims and outcomes

This study simulated trial data to examine whether adaptive designs (specifically arm dropping and stopping for futility) and the number of interim analyses in an adaptive design affects efficiency and key performance indicators of cRCTs seeking to optimise implementation strategies. The performance indicators assessed were power, the type 1 error rate, and adaptive design decisions (which arms, if any, were dropped and whether the trial stopped for futility). These results were examined over a range of trial properties reminiscent of the properties and designs commonly found in trials aiming to optimise implementation strategies.

### Data generating mechanisms

A hypothetical implementation trial using a four-arm cluster randomised control design was simulated. Entire clusters were randomised at once. Of the four arms, arm one was a control, and arms two, three, and four were treatment arms. Simulations varied in trial properties for ICC (ICC = 0.05, 0.2), number of participants per cluster (*n* = 10, 25, 50), and the number of clusters per arm (if no arms are dropped) (k = 15, 25). A fully factorial combination of these trial properties was simulated. The list of trial properties, as well as the justification for each property, is detailed in Table 1.

**Table 1:** Trial properties along with justification

Trial property	Level	Justification
ICC	0.05	A moderate ICC observed in implementation trials [31].
	0.2	A larger ICC plausible for implementation trials [10].
Number of clusters per arm	15	Suitable for a moderate to large trial [9].
	25	Plausible for a large-scale trial [32, 33].
Number of participants per cluster	10	Reflective of a community health setting such as a general practice [34].
	25	Reflective of a centre setting such as a school classroom [8].
	50	Plausible in a larger centre setting such as school grades [9].

Let $\:{Y}_{ikj}=0\:$represent an undesirable outcome and $\:{Y}_{ikj}=1$ represent a desirable outcome for the *ith* participant in the *kth* cluster in the *jth* arm. $\:{Y}_{ikj}$ was simulated following a Bernoulli distribution, with the probability of the desirable event denoted by $\:{p}_{ikj}$:

$$\:{Y}_{ikj}\sim\:Bernoulli\left({p}_{ikj}\right)$$

The probability of the favourable event was simulated on the logit scale, with the intercept ($\:{\beta\:}_{0}$), arm ($\:{X}_{i2}$, $\:{X}_{i3}$, $\:{X}_{i4}$), treatment effects ($\:{\beta\:}_{2}$, $\:{\beta\:}_{3}$, $\:{\beta\:}_{4}$), cluster ($\:{W}_{ik}$), and cluster effect variance ($\:{\alpha\:}_{k}$) being used to calculate the probability:

$$\:{p}_{ikj}=\frac{1}{1+{e}^{-({\beta\:}_{0}+{{{W}_{ik}\alpha\:}_{k}+X}_{i2}{\beta\:}_{2}+{X}_{i3}{\beta\:}_{3}+{X}_{i4}{\beta\:}_{4})}}$$

The cluster effect variance ($\:{\alpha\:}_{k}$) was calculated using the ICC in the following Eq. [35]:

$$\:{\alpha\:}_{k}=\sqrt{\frac{ICC*\frac{{\pi\:}^{2}}{3}}{1-ICC}}$$

There were two scenarios tested over the trial property space: a null scenario and an effect scenario (Table 2). In the effect scenario, Arm 4 had the highest effect and thus was the optimal arm in the trial. In the effect scenario, the probability of the favourable event ($\:{Y}_{ikj}$ = 1) occurring in Arms 4, 3, 2 and 1 (Control) were 0.4, 0.3, 0.2, and 0.1 respectively. In the null scenario, the probability of the favourable event ($\:{Y}_{ikj}$ = 1) occurring was 0.1 for all arms.

**Table 2:** Probability of favourable events by arm and scenario

	Probability of favourable event ($\:{Y}_{ikj}\:$= 1)	
Arm	Effect Scenario	Null Scenario
Control (Arm 1)	0.1	0.1
Arm 2	0.2	0.1
Arm 3	0.3	0.1
Arm 4	0.4	0.1

### Estimand

Let $\:\theta\:$ represent the log-odds ratio of the favourable outcome (Y = 1) vs. the unfavourable outcome (Y = 0).

### Simulation

Bayesian hierarchical models were run in Stan through R and the ‘cmdstanr’ package [36–38]. The priors used for treatment arm parameters $\:{\theta\:}_{j}$ for $\:j=1,\:2,\:3,\:4$, and the hyperprior specification for cluster parameters $\:{\alpha\:}_{k}$ are defined below. The treatment arm parameters $\:{\theta\:}_{j}$ for $\:j=1,\:2,\:3,\:4$ used priors that were normally distributed with a mean of 0 and standard deviation of two:

$$\:{\{{\theta\:}_{1},\theta\:}_{2},{\theta\:}_{3},{\theta\:}_{4}\}\:\sim\:N\left(0,\:2\right)$$

The cluster parameters $\:{\alpha\:}_{k}$ used a prior that was normally distributed with a mean of 0 and standard deviation of $\:{\sigma\:}_{\alpha\:}$:

$$\:{\alpha\:}_{k}\:\sim\:N\left(0,{{\upsigma\:}}_{{\upalpha\:}}\right)$$

Where $\:{\sigma\:}_{\alpha\:}$ used a hyperprior was that distributed on the half-normal distribution with a mean of 0 and standard deviation of 0.4:

$$\:{\sigma\:}_{\alpha\:}\:\sim\:half\text{-}Normal(0,\:0.4)$$

The complete Bayesian model specification was:

$$\:{Y}_{ikj}\:\sim\:Bernoulli\left({p}_{ikj}\right)$$

Where $\:{p}_{ikj}$ was defined as:

$$\:\text{ln}\left(\frac{{p}_{ikj}}{1-{p}_{ikj}}\right)=\:{\theta\:}_{1}+{{W}_{ik}\alpha\:}_{k}+{{{X}_{i}}_{2}\theta\:}_{2}+{{X}_{i}}_{3}{\theta\:}_{3}+{{X}_{i}}_{4}{\theta\:}_{4}$$

Where $\:{{\uptheta\:}}_{1}$ is the parameter corresponding to the reference group (arm one) and is equivalent to the intercept parameter $\:{{\upbeta\:}}_{0}$ in the GLM framework, $\:{X}_{2},{X}_{3},{X}_{4}$ are dichotomous variables indicating membership to treatment arms two, three, and four, respectively, and W is a matrix with K columns of dichotomous variables indicating membership to the *k*th cluster.

For each model four chains were used, with 750 warmups and 750 draws per chain, totalling 3,000 warmups and 3,000 draws. Convergence of the simulations were assessed, with acceptable convergence indicated via $\:\widehat{R}<1.05$ (measuring how well the chains agree, with values above 1 indicating disagreement) and the effective sample size (ESS) (measuring uncertainty in the estimates, lower is more uncertain) in the bulk and tail of the posterior distribution >100 per chain [37, 39].

### Number of repetitions

Each trial property combination was simulated 2,500 times. With 2,500 simulations, in a worst-case scenario of 50% power, the Monte Carlo standard error, that is, the standard error between simulations, would be 0.01 or 1% power [29] (Additional File 1).

### Adaptions

A fixed trial design and two adaptive designs were tested. The adaptive designs had either one interim analysis or two. Both adaptive designs allowed early stopping for futility and dropping the worst performing treatment arm. All outcome data for currently randomised arms were assumed to be present at the time of the interim analysis.

For each treatment arm (i.e. arms two, three and four), the posterior probability of success was defined as the proportion of posterior draws where that treatment arm’s effect estimate ($\:{\theta\:}_{j}$) was the highest:

$$\:\text{P}\text{r}({\theta\:}_{j}=\text{m}\text{a}\text{x}\{{\theta\:}_{1},{\theta\:}_{2},{\theta\:}_{3},{\theta\:}_{4}\left\}\right|data)$$

A treatment arm was eligible for dropping if its posterior probability of success was < 0.05. Only one arm could be dropped at a time, and the control arm could not be dropped. If more than one treatment arm was eligible for dropping, then the treatment arm with the lowest posterior probability of success was dropped. If more than one treatment arm had the lowest posterior probability of success (i.e. they were tied), then the treatment arm with the lowest treatment effect estimate ($\:{\theta\:}_{j}$) was dropped. The results for dropped arms were used in the later interim models and the final model to assess which arm had the largest treatment effect.

If all treatment arms had a posterior probability of success < 0.15 then the trial would stop early and conclude futility. The criteria for early stopping can expressed as:

$$\:\text{P}\text{r}({\theta\:}_{2}=\text{m}\text{a}\text{x}\{{\theta\:}_{1},{\theta\:}_{2},{\theta\:}_{3},{\theta\:}_{4}\left\}\right|data)<0.15\:\&\:$$

$$\:\text{P}\text{r}({\theta\:}_{3}=\text{m}\text{a}\text{x}\{{\theta\:}_{1},{\theta\:}_{2},{\theta\:}_{3},{\theta\:}_{4}\left\}\right|data)<0.15\:\&\:$$

$$\:\text{P}\text{r}({\theta\:}_{4}=\text{m}\text{a}\text{x}\{{\theta\:}_{1},{\theta\:}_{2},{\theta\:}_{3},{\theta\:}_{4}\left\}\right|data)<0.15$$

If a treatment arm had been dropped in an earlier interim, then only the remaining treatment arms needed the posterior probability of success < 0.15.

The control arm received a fixed number of clusters in the adaptive trials, unless the trial stopped early for futility. The clusters were equally allocated per interim. Table 3 details the allocation of clusters in the trials, assuming no arms are dropped. For trials with one or two interim analyses, the interim analysis was performed when the trial recruited approximately half or a third of the final cluster number, respectively. The effect of the timing of interims was also tested on the two interim trials. The first and second interim analyses for these trials was conducted when approximately half and three quarters of the clusters had been recruited (Table 3). These trials are referred to as the late-start trials, and their results are detailed in Additional File 2.

**Table 3:** Number of clusters allocated at each interim, assuming no arms are dropped in the trial, by number of interims, interim timing, and clusters per arm

			Number of clusters per arm in:		
N interims	Interim timing	Clusters per arm	Stage One	Stage Two	Stage Three
One	Equal spread of clusters	15	7	8	NA
		25	12	13	NA
Two	Equal spread of clusters	15	5	5	5
		25	8	8	9
	Late interims	15	7	4	4
		25	12	7	6

If a treatment arm was dropped, then the clusters that would have been assigned to the dropped treatment arm were instead equally assigned to the remaining treatment arms. If there were left over clusters after equally assigning the remaining treatment arms, then those clusters were randomly assigned to a treatment arm.

### Performance measures

Both the fixed and adaptive designs used the same calculations for the performance measures. The performance measures of interest were the power and type 1 error rate. For each trial property combination, the power was calculated as:

1 $$\:\frac{\sum\:_{1}^{{n}_{sim}}\text{t}\text{r}\text{i}\text{a}\text{l}\:\text{s}\text{u}\text{c}\text{c}\text{e}\text{s}\text{s}}{{n}_{sim}}$$

In the effect scenario, a trial was successful if the proportion of posterior draws that had $\:{\theta\:}_{4}$ as the largest treatment effect was $\:\ge\:\:$0.85:

2 $$\:P\left({\theta\:}_{4}=max\left\{{\theta\:}_{1},{\theta\:}_{2},{\theta\:}_{3},{\theta\:}_{4}\right\}\right|data)\ge\:0.85$$

In the null scenario, a trial was declared a success (albeit incorrectly) if the proportion of posterior draws that had $\:{\theta\:}_{2},\:{\theta\:}_{3},$ or $\:{\theta\:}_{4}$ as the largest treatment effect was $\:\ge\:\:$0.85. That is, any treatment arm could have a posterior probability of success $\:\ge\:$ 0.85 as defined below:

3 $$\:P\left({\theta\:}_{2}=max\left\{{\theta\:}_{1},{\theta\:}_{2},{\theta\:}_{3},{\theta\:}_{4}\right\}\right|data)\ge\:0.85\:\text{o}\text{r}\:$$

$$\:P\left({\theta\:}_{3}=max\left\{{\theta\:}_{1},{\theta\:}_{2},{\theta\:}_{3},{\theta\:}_{4}\right\}\right|data)\ge\:0.85\:\text{o}\text{r}$$

$$\:P\left({\theta\:}_{4}=max\left\{{\theta\:}_{1},{\theta\:}_{2},{\theta\:}_{3},{\theta\:}_{4}\right\}\right|data)\ge\:0.85$$

To calculate the type 1 error rate, the same equation for calculating power (Eq. 1) was used, except it was based on the number of incorrectly declared trial successes in the null scenario.

In the effect scenario, the scaled power was also calculated. The scaled power is the power when the type 1 error rate is fixed at 0.05. This power was calculated as the proportion of successful trials (Eq. 1), but the threshold used to determine whether the trial was successful (Eq. 2) instead of 0.85 was equal the threshold that resulted in a type 1 error rate of 0.05 in the corresponding null scenario (Eq. 3).

The power and type 1 error rate were compared between the zero interim (fixed), one interim and two interim designs over the trial property space.

### Sensitivity analyses

To determine the effect of the choice of seed or prior on the results a random subset of the simulations with adaptive designs were rerun (2,500 repetitions) with a different seed, or vague priors for the treatment effects, respectively. These priors for the treatment effects used a normal distribution with a mean of 0 and standard deviation of 100:

$$\:{\{{\theta\:}_{1},\:\theta\:}_{2},{\theta\:}_{3},{\theta\:}_{4}\}\:\sim\:N\left(\text{0,100}\right)$$

To determine if the effect of the candidate adaptive design was consistent in a trial with a less clear optimal arm, the simulations were run with the following favourable event probabilities as a sensitivity analysis: Arm One (Control) = 0.1; Arm Two = 0.1; Arm Three = 0.15; Arm Four = 0.2. The results for this sensitivity analysis are tabulated in Additional File 2.

### Case study

To assess how the efficiency and applicability of the candidate adaptive design compares to sequential and fixed trials from a real-world scenario, we conducted a case study. We considered two previously completed cRCTs by Nathan [7] and Lane [6] that aimed to increase the minutes of physical activity teachers delivered to students in schools. These trials were delivered sequentially and aimed to optimise an implementation strategy promoting the delivery of physical activity by teachers. We used a binary version of the primary outcomes in these trials: whether the teachers delivered 150 + minutes of physical activity to students per week. Dichotomising the primary outcome is expected to reduce the power compared to the original studies, but this is reasonable since the case study is for illustrative purposes. The first trial [7] found that the experimental strategy was superior to usual care, and the second trial [6] found that an updated version of the strategy was noninferior to the original strategy. For the case study, we used a superiority framework instead of the original noninferiority framework from the second trial. All four arms were included, with the original strategy arms from the two trials treated as distinct arms for the purposes of comparison.

We compared three designs that these trials could have used: (1) a series of up to three sequential, fixed, two-arm cRCTs; (2) a four-arm, fixed cRCT; (3) a four-arm, adaptive cRCT that allowed for early stopping for futility and/or dropping the worst-performing treatment arm for futility with two interim analyses. While the number of clusters and number of participants per cluster differed in the trials, we assumed an equal number of clusters and participants per cluster. We set the number of clusters per arm to 31, the participants per cluster to 6 (reflective of the usual care arm in the first trial), and the ICC to 0.07. The proportion of teachers who delivered 150 + minutes of physical activity to students per week was 0.172 in the usual care arm, 0.619 in the original strategy from the first trial, 0.649 in the original strategy from the second trial, and 0.692 in the updated strategy.

These case study trials followed the previously stated calculations and rules in the Methods section. The sequential trials progressed through three comparisons (totalling 6 arms): first, usual care was compared to the original strategy from the first trial; next, the original strategy from the first trial was compared to the original strategy from the second trial; and finally, the original strategy from the second trial was compared to the updated strategy. Each subsequent trial was conducted only if the preceding trial concluded that the newer strategy was more effective than the comparator. We considered two scenarios: (1) a scenario where the original trials’ proportions were observed; (2) a scenario where the proportion of teachers delivering 150 + minutes of physical activity per week to students was 0.172 in all arms. The first scenario was used to determine power, and the second scenario was used to determine the type 1 error rate. Scaled power was calculated for the two four-arm trials, but not for the sequential trials. Calculating scaled power would require information from later trials, specifically, data that would only exist once the threshold for success had already been applied.

## Results

All results are tabulated in Additional File 2. To help readers who are new to adaptive designs, examples of single adaptive trials that were simulated are presented in Additional File 3.

Across both the null and effect scenarios, convergence was acceptable: less than 5% of trials had an $\:\widehat{R\:}$>1.05, an ESS bulk < 400, or ESS tail < 400, indicating acceptable agreement between chains and measurement uncertainty, respectively. One trial property combination (Effect scenario, ICC = 0.2, number of clusters = 25, number of participants per cluster = 50) had approximately 10% of simulations with an ESS bulk between 400 and 300 for the one and two interim adaptive designs.

**Fig. 1:**
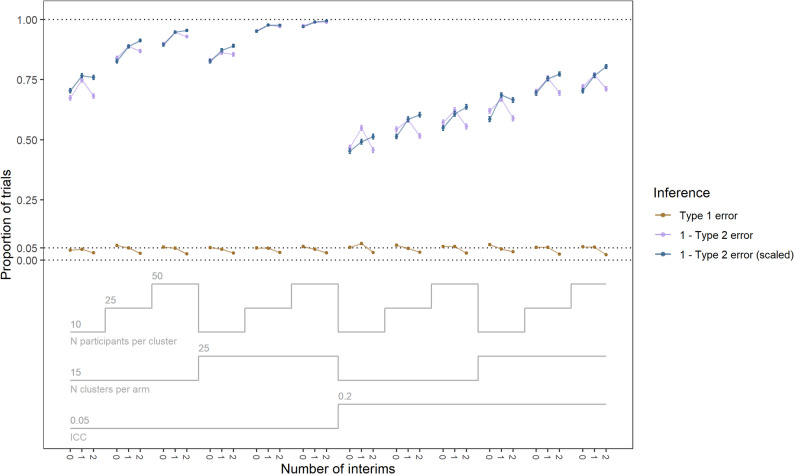
Power (1-Type 2 error) and type 1 error rate of the fixed and adaptive designs with 0 (fixed), 1, and 2 interims over trial properties. The scaled power is the power corresponding to a fixed type 1 error rate of 0.05. The unscaled power is the power relating to the un-fixed type 1 error rate. ICC = intra-class correlation

The scaled power was consistently higher, and the type 1 error was consistently lower with an increasing number of interims (Fig. 1). The unscaled power ranged from 0.47 to 0.97 for zero interim trials, 0.55 to 0.99 for one interim trials, and 0.45 to 0.99 for two interim trials. The type 1 error rate ranged from 0.041 to 0.064 for zero interim trials, 0.045 to 0.068 for one interim trials, and 0.022 to 0.034 for two interim trials. The scaled power was almost always higher with an increasing number of interims. The scaled power ranged from 0.45 to 0.97 for zero interim trials, 0.49 to 0.99 for one interim trials, and 0.51 to 0.99 for two interim trials. The type 1 error rate ranged from 0.022 to 0.039 for the late-start two interim trials (Additional File 2 - Additional Table 4). The unscaled and scaled power for the late-start two interim trials ranged from 0.47 to 0.99 and 0.51 to 0.995, respectively. The unscaled power was comparable to higher in the late start trials compared to the main two interim trials.

**Fig. 2:**
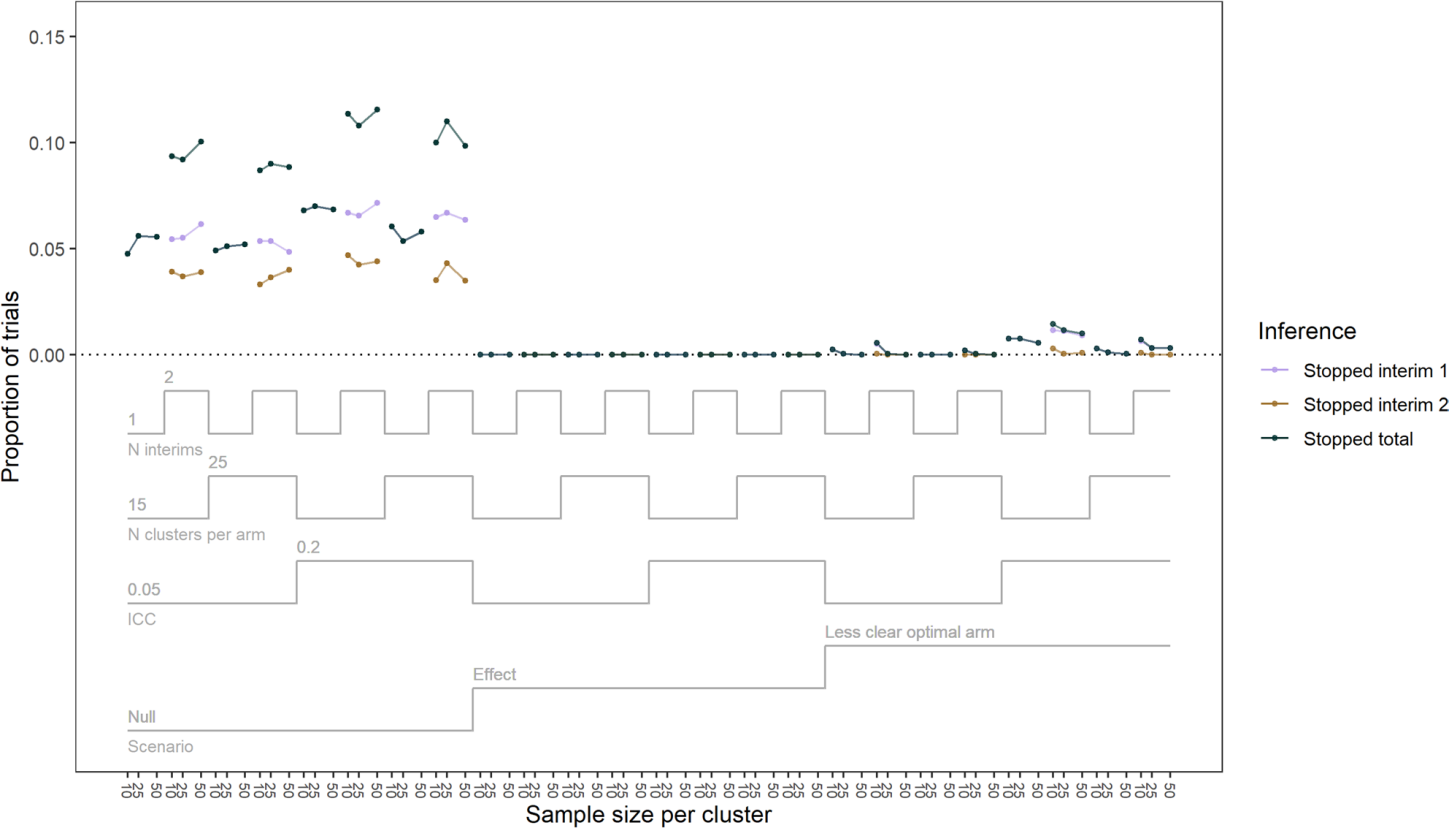
Proportion of trials that stopped for futility over trial properties and 1 or 2 interims. ICC = intra-class correlation

No trials stopped for futility in the effect scenario with one or two interims (Fig. 2). In the null scenario at interim one, the one and two interim trials stopped for futility 4.8 to 7.0% and 4.8 to 7.2% of the time, respectively. Then, a further 3.3 to 4.7% of trials stopped for futility at interim two (8.7 to 11.6% total at end of trial) for the two interim design. Slightly more trials stopped for futility in the null scenario when the ICC was high and number of clusters was lower. There was no clear pattern between the number of participants per cluster and the proportion of trials that stopped for futility in the null scenario. In the late-start trials, 7.2 to 11.0% of the trials stopped for futility in the null scenario, and 0% in the effect scenario (Additional File 2 - Additional Table 5).

**Fig. 3:**
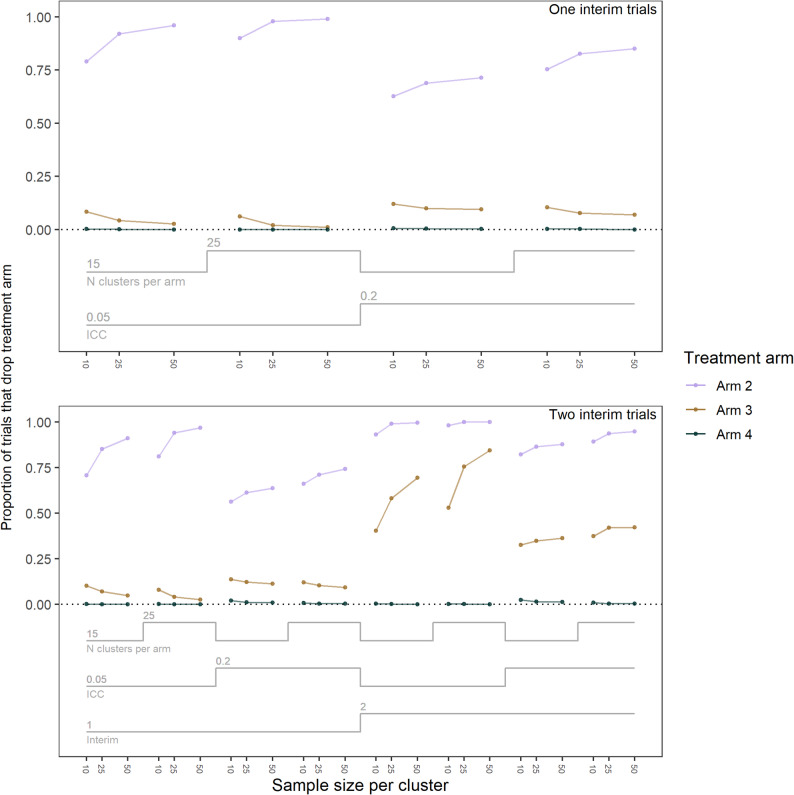
Proportion of trials that dropped a treatment arm by number of interims and trial properties in the effect scenario. The top graph presents trials with only one interim and presents what treatment arms were dropped at interim one. The bottom graph presents trials with two interims and presents what treatment arms were dropped at interim one (left) and then at interim two (right). ICC = intra-class correlation

For the effect scenario at interim one, in both the one interim and two interim trials, Arm two was dropped in 56.2 to 98.9% of trials, Arm three was dropped in 1.0 to 13.6% of trials, and Arm four in 0 to 2.0% of trials (Fig. 3). At interim two, Arm two had been dropped in 82.1 to 100% of trials, Arm three in 32.6 to 84.4% of trials, and Arm four in 0 to 2.4% of trials. For the late-start trials in the effect scenario, by interim two, Arm Two was dropped in 83.1 to 100% of trials, Arm Three in 33.2 to 88.2% of trials, and Arm Four in 0 to 1.0% of trials (Additional File 2 - Additional Table 6).

Across the one and two interim trials, by the end of the trial Arm two was dropped less frequently, and Arm four more frequently when the ICC was higher and when the number of clusters per arm was lower. When the ICC was higher and by the end of the trial, Arm three was dropped more frequently in the one interim trials, but less frequently in the two interim trials. By the end of the trial, the two interim trials dropped all arms more frequently than the one interim trials.

In the null scenario by the end of the trial, all arms were dropped more frequently in the trials with two interims (Additional File 4).

### Sensitivity analysis

Neither the reported power nor type 1 error rate differed by more than 1% in trials when using an uninformative prior compared to the main analysis. At most, 2.5% more trials had an ESS bulk < 400 with the uninformative prior, but the proportion of trials with an ESS tail < 400 or $\:\widehat{R\:}$>1.05 didn’t differ by more than 0.5%. When using a different seed, neither the power, type 1 error rate, nor convergence characteristics differed by more than 1% compared to the main analysis.

In the effect scenario where there was a less clear optimal arm, the scaled power and type 1 error rate for the fixed trials ranged from 0.27 to 0.87 and 0.041 to 0.064, respectively (Additional File 2 – Additional Table 7). For the adaptive trials, the scaled power and type 1 error rate ranged from 0.29 to 0.94, and 0.022 to 0.068, respectively. The effect of the adaptive designs and the number of interim analyses across the trial properties on the power and type 1 error rate was consistent with the main analysis. Having an extra interim, a higher ICC, fewer clusters, and fewer participants per cluster increased the risk of incorrectly stopping for futility or incorrectly dropping the most effective arm (Fig. 2).

### Case study

In the sequential trials, the power was 0.123 and the type 1 error rate was 0.016 (Table 4). The fixed trial had a power of 0.282 and a type 1 error rate of 0.0564. For the adaptive trial, the power was 0.241 with a type 1 error rate of 0.0268. When the power was scaled to a type 1 error rate of 0.05, the adaptive trial had a scaled power of 0.31 while the scaled power was 0.268 in the fixed trial. In the null scenario, the mean sample size was 446 for the sequential trials and 692 for the adaptive trial. In the effect scenario, the mean sample size in the sequential trials was 138 participants higher than the fixed and adaptive trials.

**Table 4:** Power, type 1 error rate, scaled power, and mean sample size (in the null scenario) by trial design (sequential two-arm cRCTs, fixed four-arm cRCT, or adaptive four-arm cRCT)

Design	Power	Type 1 error rate	Scaled power	Mean sample size (null scenario)	Mean sample size (effect scenario)	Maximum sample size
Sequential	0.123	0.016	NA	446	882	1116
Fixed	0.282	0.0564	0.268	744	744	744
Adaptive	0.241	0.0268	0.310	692	744	744

## Discussion

We compared the power and type 1 error rate in four-arm cRCTs with either a fixed design or an adaptive design with one or two interim analyses across a range of trial properties. In addition, we compared the adaptive design decisions made (arm dropping, early stopping for futility) between trials with one and two interim analyses.

The adaptive designs offered scaled power increases and a lower type 1 error rate compared to the fixed designs. Across most trial properties, the adaptive trials with one interim analysis had a slightly lower scaled power than the trials with two interim analyses. Our results align with those from Ryan et al., that saw power and type 1 error rate differences between one and two interims, with the power and type 1 error rate changing gradually over an increasing number of interim analyses [20]. However, the simulation by Ryan et al. did not include arm dropping, which may explain why our simulation saw a larger power increase between a fixed design and an adaptive design with one interim analysis. The decreased type 1 error rate in our simulation for the one and two interim analyses translated into scaled power gains. When preparing for a trial, it is recommended to determine the cut points for decisions rules by performing a simulation that reflects the expected properties of the trial to calculate a cut point that results in an acceptable type 1 error rate [28]. It is important to note that our rule to determine trial success in the simulations aligns with the aim of optimisation. Specifically, the trial was only successful only if it identified the true optimal arm, not any arm other than control. Optimisation seeks to find the optimal treatment (or combination of treatment components) within given constraints. We recommend that trials aiming to optimise are adequately powered to determine which arm is optimal, rather than detecting that any arm is better than control.

Trials with two interim analyses correctly stopped for futility approximately two times as often as one interim trials. The adaptive trials more frequently stopped for futility when the number of clusters was lower and the ICC was higher. Trials with a lower number of clusters or a higher ICC had a higher variance in the model estimates, which in turn increased the proportion of trials that stopped for futility. The two interim trials stopped at interim one approximately the same amount as the one interim trials. However, because the two interim trials’ first interim analysis were at 33% of the clusters, it correctly stopped earlier than the one interim trials, which had their interim at 50% clusters. The two interim design, because of its ability to more readily and rapidly declare futility, offers reduced resource wastage when conducting trials that are ineffective. The late-start two interim trials had a comparable to slightly lower type 1 error rate and a higher scaled power compared to the main two interim trials. The less effective arms were also dropped more often in the late start trials. The slightly lower type 1 error and more frequent dropping of ineffective arms could be due more data being available at the first interim analysis, which could increase the likelihood of making a correct interim design decision.

The additional interim increased the rate at which ineffective arms would be dropped. The two interim trials dropped the least effective arm more frequently when the variance was lower i.e. when the ICC was lower, there were more clusters, and more participants per cluster. The increased rate of arm Three being dropped in the two interim trials was likely due to the restriction of one arm being dropped per interim. It can be argued that potentially dropping ineffective arms sooner is more ethically sound to the participants (as they are more likely to receive a more effective treatment) [40] or can improve recruitment as the design is more appealing to a potential participant [18]. However, whether the improved ethical stance remains when there is a higher chance of dropping an effective arm (when variance is higher) is questionable. Although there is not a clear argument for arm dropping from an ethical standpoint in this context, there is sound statistical reasoning. While dropping an arm for futility can increase the variance in that arm’s effect estimate, it also increased the precision of the estimates for the remaining treatment arms. This is because more clusters are then randomised to the remaining treatment arms. By diverting clusters away from futile arms and towards those showing more promise, we are better able to estimate their true treatment effects.

A higher ICC led to the trials performing worse. It resulted in a lower scaled and unscaled power, dropping more effective arms more frequently, and incorrectly stopping for futility. The incorrect decisions when the ICC was high was compounded when there were two interims and the first interim was at 33% through the trial. The incorrect decisions from the high ICC and two interims were lessened when the first interim was 50% through the trial, but weren’t avoided completely. This suggests that the incorrect decisions are partially due to the earlier interim decisions (at 33% through the trial), but also partially due to the multiplicity of the interim decisions. Due to these results, we recommend that if researchers are using two interims, then they should consider whether first interim should start later in the trial if they suspect the outcome of interest will have a high ICC. Systematic reviews on implementation trials have reported median ICC values consistently less than 0.1 [31, 41] meaning it is unlikely that implementation trials will encounter these adaptive design limitations, but outcomes highly dependent on cluster level strategies (e.g. class time physical activity) are still at risk of a high ICC values [10]. Whilst a lower number of clusters and participants per cluster resulted in slightly worse performance of the adaptive designs, the performance was not impacted as much as the ICC. Even if recruitment is challenging, or if the effective size is smaller than expected (thus reducing power), it is acceptable to include an additional interim as the scaled power is not adversely affected, and the increased risk of incorrect adaptive design decisions are minimal.

In this simulation, we considered a maximum of two interims as implementation trials are already complex, and additional interims may be unmanageable. Implementation outcomes can require significant resources to measure and are dependent on the length of time required to obtain the outcome of interest [34, 42]. For example, if the outcome is obtained at 12 months, each interim analysis will add an additional 12 months onto the length of the trial. In addition, recruitment may need to be paused while the interim analysis is being conducted, further extending the length of the trial, and the additional workload from the trial statistician can increase the trial cost [43]. This downside can potentially be alleviated by including a proxy outcome, such as the outcome measured early at 6 months. However, if the proxy outcome is not a perfect predictor of the main outcome, then the adaptive designs decisions are more likely to be incorrect.

In addition to the simulations, we performed a case study assessing the properties of candidate designs using previously conducted trials. In the comparisons between the fixed and adaptive designs, the adaptive designs had a higher scaled power than the fixed design, mainly due to the lower type 1 error rate, which is consistent with our simulations. In the null scenario, the sequential trials had the lowest type 1 error rate and mean sample size. This is expected as each trial had to conclude success before the next trial could proceed, meaning that the multiple comparisons were restricted by the success of the prior trials. This restriction also explains the lower power in the sequential trials compared to the four-arm trials. In the effect scenario, the sequential trials had a higher mean sample size compared to the four-arm trials. This is partly because identifying the optimal strategy using sequential trials requires at least 6 arms (total sample size of 1116), compared to four in the multi-arm trials (total sample size of 744). The case study supports the simulation findings that adaptive designs can offer efficiency gains over fixed designs.

### Limitations

Our simulation study was not without limitations. We tested a large and smaller treatment effect, but how these designs perform with other combinations of treatment effects is unknown. It is likely that the benefits of an addition interim analysis will be attenuated with an even smaller treatment effect. To address the increased risk of making an incorrect adaptive design decision (i.e. dropping an effective arm or stopping for futility when strategies are effective) with smaller treatment effects, the cut points for these decisions may need to be more conservative. In turn however, more conservative cut points will cause a trial to stop for futility and drop the least effective arm less often.

The simulation assumed no missing data, which is unlikely for large trials. Missing data will decrease the usefulness of the adaptive designs, and if the data are missing not at random, may increase the bias of estimates [44] leading to increasing the likelihood of the incorrect decision being made such as keeping an ineffective arm. It is imperative that all efforts are made to obtain outcome data, and if that is not possible, discern why the data are missing (e.g. treatment failure, constraints too high, or missing at random), or consider imputation of the missing data [45].

## Conclusion

Both one and two interim adaptive designs offered scaled power increases compared to fixed designs. Across most trial properties two interim designs had a higher scaled power than the one interim designs. Designs with two interim analyses were more efficient than the one interim and fixed designs. Adaptive designs with two interim analyses and an earlier first interim analysis are recommended for trials with a low ICC on the main outcome. If the ICC is expected to be high then it may be preferable to delay the first interim analysis to later in the trial.

## Supplementary Information

Supplementary Material 1. Additional File 1, Calculation to determine number of simulations needed.

Supplementary Material 2. Additional File 2, Tabulated results for simulations, including the main results, the results from the delayed interim trials, and the results from the unclear optimal arm scenario

Supplementary Material 3. Additional File 3, Examples of adaptive trials

Supplementary Material 4. Additional File 4, Proportion of trials that dropped a treatment arm by number of interims and trial properties in the null scenario. ICC = intra-class correlation

## Data Availability

Code for the simulations is located at [https://github.com/ErnKNolan/PhDProj3] (https://github.com/ErnKNolan/PhDProj3). Simulated datasets are located at [https://osf.io/kj7yf/] (https://osf.io/kj7yf). Results were generated using R version 4.3.1, cmdstanr version 0.6.1, and Stan version 2.26.1.

## Abbreviations

ADEMP: Aims, data generating mechanisms, estimands, methods, performance measures
cRCT: Cluster randomised control trial
ESS: Effective sample size
ICC: Intra-class correlation
MAMS: Multi-arm multi-stage
RCT: Randomised control trial
