# Supplementary material for: Adaptive designs for trials aiming to optimise implementation strategies and the effect of an additional interim analysis: a simulation study
Source: BMC Med Res Methodol. 2025 Nov 29;26:1. doi: 10.1186/s12874-025-02730-y (PMC12771792; doi:10.1186/s12874-025-02730-y)
Supplement: Supplementary file 1 — Supplementary Material 1. Additional File 1, Calculation to determine number of simulations needed. [file 12874_2025_2730_MOESM1_ESM.docx]

Additional File 1: Calculation to determine number of simulations needed.

$$\frac{\hat{power}*(1-\hat{power})}{MCSE^{2}}=n_{sim}$$

$$\frac{0.5\cdot0.5}{{0.01}^{2}}=2500$$
