# Supplementary material for: Adaptive designs for trials aiming to optimise implementation strategies and the effect of an additional interim analysis: a simulation study
Source: BMC Med Res Methodol. 2025 Nov 29;26:1. doi: 10.1186/s12874-025-02730-y (PMC12771792; doi:10.1186/s12874-025-02730-y)
Supplement: Supplementary file 2 — Supplementary Material 2. Additional File 2, Tabulated results for simulations, including the main results, the results from the delayed interim trials, and the results from the unclear optimal arm scenario [file 12874_2025_2730_MOESM2_ESM.docx]

**Additional File 2: Tabulated results for simulations, including the main results, the results from the delayed interim trials, and the results from the unclear optimal arm scenario.**

*Additional Table 1:* *Power (1-Type 2 error) and type 1 error of the fixed and adaptive designs with 0 (fixed), 1, and 2 interims over trial properties.*

|  |  |  |  | Proportion (±1 Monte Carlo Standard Error) | | |
| --- | --- | --- | --- | --- | --- | --- |
| N interims | ICC | N clusters per arm | N participants per cluster | Type 1 error | 1 - Type 2 error | 1 - Type 2 error (scaled) |
| 0 | 0.05 | 15 | 10 | 0.041 (0.037, 0.045) | 0.674 (0.665, 0.683) | 0.704 (0.695, 0.714) |
| 1 | 0.05 | 15 | 10 | 0.045 (0.041, 0.049) | 0.749 (0.74, 0.757) | 0.766 (0.758, 0.775) |
| 2 | 0.05 | 15 | 10 | 0.03 (0.026, 0.033) | 0.682 (0.672, 0.691) | 0.759 (0.751, 0.768) |
| 0 | 0.2 | 15 | 10 | 0.052 (0.048, 0.057) | 0.467 (0.457, 0.477) | 0.453 (0.443, 0.463) |
| 1 | 0.2 | 15 | 10 | 0.068 (0.063, 0.073) | 0.549 (0.539, 0.559) | 0.492 (0.482, 0.502) |
| 2 | 0.2 | 15 | 10 | 0.031 (0.027, 0.034) | 0.457 (0.447, 0.467) | 0.513 (0.503, 0.523) |
| 0 | 0.05 | 25 | 10 | 0.051 (0.047, 0.056) | 0.83 (0.822, 0.838) | 0.826 (0.819, 0.834) |
| 1 | 0.05 | 25 | 10 | 0.045 (0.041, 0.049) | 0.862 (0.856, 0.869) | 0.872 (0.866, 0.879) |
| 2 | 0.05 | 25 | 10 | 0.029 (0.025, 0.032) | 0.855 (0.848, 0.862) | 0.89 (0.884, 0.897) |
| 0 | 0.2 | 25 | 10 | 0.064 (0.059, 0.069) | 0.62 (0.61, 0.63) | 0.586 (0.576, 0.595) |
| 1 | 0.2 | 25 | 10 | 0.046 (0.041, 0.05) | 0.669 (0.659, 0.678) | 0.686 (0.677, 0.695) |
| 2 | 0.2 | 25 | 10 | 0.034 (0.03, 0.038) | 0.589 (0.579, 0.599) | 0.665 (0.656, 0.675) |
| 0 | 0.05 | 15 | 25 | 0.06 (0.056, 0.065) | 0.84 (0.833, 0.847) | 0.827 (0.819, 0.834) |
| 1 | 0.05 | 15 | 25 | 0.05 (0.046, 0.054) | 0.888 (0.882, 0.894) | 0.888 (0.882, 0.894) |
| 2 | 0.05 | 15 | 25 | 0.028 (0.025, 0.031) | 0.868 (0.862, 0.875) | 0.913 (0.908, 0.919) |
| 0 | 0.2 | 15 | 25 | 0.062 (0.057, 0.066) | 0.544 (0.534, 0.554) | 0.514 (0.504, 0.524) |
| 1 | 0.2 | 15 | 25 | 0.048 (0.044, 0.052) | 0.582 (0.573, 0.592) | 0.585 (0.575, 0.595) |
| 2 | 0.2 | 15 | 25 | 0.032 (0.029, 0.036) | 0.516 (0.506, 0.526) | 0.604 (0.594, 0.614) |
| 0 | 0.05 | 25 | 25 | 0.05 (0.046, 0.055) | 0.952 (0.948, 0.956) | 0.951 (0.947, 0.956) |
| 1 | 0.05 | 25 | 25 | 0.049 (0.044, 0.053) | 0.977 (0.974, 0.98) | 0.977 (0.974, 0.98) |
| 2 | 0.05 | 25 | 25 | 0.032 (0.028, 0.035) | 0.97 (0.967, 0.973) | 0.976 (0.973, 0.979) |
| 0 | 0.2 | 25 | 25 | 0.052 (0.048, 0.057) | 0.701 (0.692, 0.71) | 0.694 (0.684, 0.703) |
| 1 | 0.2 | 25 | 25 | 0.053 (0.048, 0.057) | 0.757 (0.749, 0.766) | 0.754 (0.745, 0.762) |
| 2 | 0.2 | 25 | 25 | 0.024 (0.021, 0.027) | 0.694 (0.685, 0.704) | 0.773 (0.765, 0.782) |
| 0 | 0.05 | 15 | 50 | 0.054 (0.049, 0.059) | 0.898 (0.892, 0.904) | 0.895 (0.889, 0.901) |
| 1 | 0.05 | 15 | 50 | 0.05 (0.045, 0.054) | 0.947 (0.942, 0.951) | 0.948 (0.944, 0.952) |
| 2 | 0.05 | 15 | 50 | 0.025 (0.022, 0.028) | 0.929 (0.924, 0.934) | 0.954 (0.95, 0.959) |
| 0 | 0.2 | 15 | 50 | 0.056 (0.051, 0.061) | 0.572 (0.562, 0.582) | 0.549 (0.539, 0.559) |
| 1 | 0.2 | 15 | 50 | 0.056 (0.051, 0.06) | 0.623 (0.613, 0.632) | 0.608 (0.598, 0.618) |
| 2 | 0.2 | 15 | 50 | 0.029 (0.025, 0.032) | 0.555 (0.545, 0.565) | 0.636 (0.627, 0.646) |
| 0 | 0.05 | 25 | 50 | 0.056 (0.051, 0.061) | 0.974 (0.971, 0.978) | 0.971 (0.967, 0.974) |
| 1 | 0.05 | 25 | 50 | 0.045 (0.041, 0.049) | 0.988 (0.986, 0.991) | 0.989 (0.987, 0.991) |
| 2 | 0.05 | 25 | 50 | 0.03 (0.027, 0.034) | 0.988 (0.986, 0.99) | 0.994 (0.992, 0.995) |
| 0 | 0.2 | 25 | 50 | 0.055 (0.051, 0.06) | 0.72 (0.711, 0.729) | 0.704 (0.695, 0.713) |
| 1 | 0.2 | 25 | 50 | 0.053 (0.049, 0.058) | 0.77 (0.762, 0.779) | 0.767 (0.758, 0.775) |
| 2 | 0.2 | 25 | 50 | 0.022 (0.019, 0.025) | 0.712 (0.703, 0.721) | 0.805 (0.797, 0.813) |

*Additional Table 2: Proportion of trials that stopped for futility over trial properties and 1 or 2 interims. ICC = intra-class correlation.*

|  |  |  |  |  | Proportion | | |
| --- | --- | --- | --- | --- | --- | --- | --- |
| Scenario | N Interims | ICC | N clusters per arm | N participants per cluster | Stopped interim 1 | Stopped interim 2 | Stopped total |
| Null | 1 | 0.05 | 15 | 10 | 0.0476 | NA | 0.0476 |
| Null | 2 | 0.05 | 15 | 10 | 0.0544 | 0.0392 | 0.0936 |
| Null | 1 | 0.2 | 15 | 10 | 0.068 | NA | 0.068 |
| Null | 2 | 0.2 | 15 | 10 | 0.0668 | 0.0468 | 0.1136 |
| Null | 1 | 0.05 | 25 | 10 | 0.0492 | NA | 0.0492 |
| Null | 2 | 0.05 | 25 | 10 | 0.0536 | 0.0332 | 0.0868 |
| Null | 1 | 0.2 | 25 | 10 | 0.0604 | NA | 0.0604 |
| Null | 2 | 0.2 | 25 | 10 | 0.0648 | 0.0352 | 0.1 |
| Null | 1 | 0.05 | 15 | 25 | 0.056 | NA | 0.056 |
| Null | 2 | 0.05 | 15 | 25 | 0.0552 | 0.0368 | 0.092 |
| Null | 1 | 0.2 | 15 | 25 | 0.07 | NA | 0.07 |
| Null | 2 | 0.2 | 15 | 25 | 0.0656 | 0.0424 | 0.108 |
| Null | 1 | 0.05 | 25 | 25 | 0.0512 | NA | 0.0512 |
| Null | 2 | 0.05 | 25 | 25 | 0.0536 | 0.0364 | 0.09 |
| Null | 1 | 0.2 | 25 | 25 | 0.0536 | NA | 0.0536 |
| Null | 2 | 0.2 | 25 | 25 | 0.0668 | 0.0432 | 0.11 |
| Null | 1 | 0.05 | 15 | 50 | 0.0556 | NA | 0.0556 |
| Null | 2 | 0.05 | 15 | 50 | 0.0616 | 0.0388 | 0.1004 |
| Null | 1 | 0.2 | 15 | 50 | 0.0684 | NA | 0.0684 |
| Null | 2 | 0.2 | 15 | 50 | 0.0716 | 0.044 | 0.1156 |
| Null | 1 | 0.05 | 25 | 50 | 0.052 | NA | 0.052 |
| Null | 2 | 0.05 | 25 | 50 | 0.0484 | 0.04 | 0.0884 |
| Null | 1 | 0.2 | 25 | 50 | 0.058 | NA | 0.058 |
| Null | 2 | 0.2 | 25 | 50 | 0.0636 | 0.0348 | 0.0984 |
| Effect | 1 | 0.05 | 15 | 10 | 0 | NA | 0 |
| Effect | 2 | 0.05 | 15 | 10 | 0 | 0 | 0 |
| Effect | 1 | 0.2 | 15 | 10 | 0 | NA | 0 |
| Effect | 2 | 0.2 | 15 | 10 | 0 | 0 | 0 |
| Effect | 1 | 0.05 | 25 | 10 | 0 | NA | 0 |
| Effect | 2 | 0.05 | 25 | 10 | 0 | 0 | 0 |
| Effect | 1 | 0.2 | 25 | 10 | 0 | NA | 0 |
| Effect | 2 | 0.2 | 25 | 10 | 0 | 0 | 0 |
| Effect | 1 | 0.05 | 15 | 25 | 0 | NA | 0 |
| Effect | 2 | 0.05 | 15 | 25 | 0 | 0 | 0 |
| Effect | 1 | 0.2 | 15 | 25 | 0 | NA | 0 |
| Effect | 2 | 0.2 | 15 | 25 | 0 | 0 | 0 |
| Effect | 1 | 0.05 | 25 | 25 | 0 | NA | 0 |
| Effect | 2 | 0.05 | 25 | 25 | 0 | 0 | 0 |
| Effect | 1 | 0.2 | 25 | 25 | 0 | NA | 0 |
| Effect | 2 | 0.2 | 25 | 25 | 0 | 0 | 0 |
| Effect | 1 | 0.05 | 15 | 50 | 0 | NA | 0 |
| Effect | 2 | 0.05 | 15 | 50 | 0 | 0 | 0 |
| Effect | 1 | 0.2 | 15 | 50 | 0 | NA | 0 |
| Effect | 2 | 0.2 | 15 | 50 | 0 | 0 | 0 |
| Effect | 1 | 0.05 | 25 | 50 | 0 | NA | 0 |
| Effect | 2 | 0.05 | 25 | 50 | 0 | 0 | 0 |
| Effect | 1 | 0.2 | 25 | 50 | 0 | NA | 0 |
| Effect | 2 | 0.2 | 25 | 50 | 0 | 0 | 0 |

*Additional Table 3: Proportion of trials that dropped a treatment arm by number of interims and trial properties in the effect scenario. ICC = intra-class correlation.*

|  |  |  |  |  | Proportion | | |
| --- | --- | --- | --- | --- | --- | --- | --- |
| Number of interims | ICC | N clusters per arm | N participants per cluster | Interim | Arm 2 | Arm 3 | Arm 4 |
| 1 | 0.05 | 15 | 10 | 1 | 0.7904 | 0.0836 | 0.002 |
| 1 | 0.05 | 15 | 10 | 2 | NA | NA | NA |
| 2 | 0.05 | 15 | 10 | 1 | 0.7076 | 0.1008 | 0.0016 |
| 2 | 0.05 | 15 | 10 | 2 | 0.9316 | 0.404 | 0.0028 |
| 1 | 0.2 | 15 | 10 | 1 | 0.626 | 0.1208 | 0.0064 |
| 1 | 0.2 | 15 | 10 | 2 | NA | NA | NA |
| 2 | 0.2 | 15 | 10 | 1 | 0.5616 | 0.1364 | 0.0196 |
| 2 | 0.2 | 15 | 10 | 2 | 0.8208 | 0.3256 | 0.024 |
| 1 | 0.05 | 25 | 10 | 1 | 0.9 | 0.062 | 0 |
| 1 | 0.05 | 25 | 10 | 2 | NA | NA | NA |
| 2 | 0.05 | 25 | 10 | 1 | 0.8104 | 0.0796 | 0.0004 |
| 2 | 0.05 | 25 | 10 | 2 | 0.9816 | 0.5288 | 0.0004 |
| 1 | 0.2 | 25 | 10 | 1 | 0.7532 | 0.1048 | 0.0024 |
| 1 | 0.2 | 25 | 10 | 2 | NA | NA | NA |
| 2 | 0.2 | 25 | 10 | 1 | 0.66 | 0.1196 | 0.0072 |
| 2 | 0.2 | 25 | 10 | 2 | 0.8924 | 0.3728 | 0.0092 |
| 1 | 0.05 | 15 | 25 | 1 | 0.9204 | 0.0428 | 0.0004 |
| 1 | 0.05 | 15 | 25 | 2 | NA | NA | NA |
| 2 | 0.05 | 15 | 25 | 1 | 0.8504 | 0.0692 | 0 |
| 2 | 0.05 | 15 | 25 | 2 | 0.9908 | 0.5804 | 0.0004 |
| 1 | 0.2 | 15 | 25 | 1 | 0.6884 | 0.1 | 0.0044 |
| 1 | 0.2 | 15 | 25 | 2 | NA | NA | NA |
| 2 | 0.2 | 15 | 25 | 1 | 0.6128 | 0.1224 | 0.0104 |
| 2 | 0.2 | 15 | 25 | 2 | 0.864 | 0.348 | 0.0148 |
| 1 | 0.05 | 25 | 25 | 1 | 0.9792 | 0.0196 | 0 |
| 1 | 0.05 | 25 | 25 | 2 | NA | NA | NA |
| 2 | 0.05 | 25 | 25 | 1 | 0.9404 | 0.0392 | 0 |
| 2 | 0.05 | 25 | 25 | 2 | 0.9988 | 0.7544 | 0.0004 |
| 1 | 0.2 | 25 | 25 | 1 | 0.8268 | 0.0776 | 0.002 |
| 1 | 0.2 | 25 | 25 | 2 | NA | NA | NA |
| 2 | 0.2 | 25 | 25 | 1 | 0.71 | 0.104 | 0.0036 |
| 2 | 0.2 | 25 | 25 | 2 | 0.9368 | 0.4192 | 0.004 |
| 1 | 0.05 | 15 | 50 | 1 | 0.9596 | 0.026 | 0 |
| 1 | 0.05 | 15 | 50 | 2 | NA | NA | NA |
| 2 | 0.05 | 15 | 50 | 1 | 0.9112 | 0.0472 | 0 |
| 2 | 0.05 | 15 | 50 | 2 | 0.996 | 0.6932 | 0 |
| 1 | 0.2 | 15 | 50 | 1 | 0.7136 | 0.094 | 0.0028 |
| 1 | 0.2 | 15 | 50 | 2 | NA | NA | NA |
| 2 | 0.2 | 15 | 50 | 1 | 0.6364 | 0.1132 | 0.0092 |
| 2 | 0.2 | 15 | 50 | 2 | 0.878 | 0.3616 | 0.0116 |
| 1 | 0.05 | 25 | 50 | 1 | 0.9892 | 0.01 | 0 |
| 1 | 0.05 | 25 | 50 | 2 | NA | NA | NA |
| 2 | 0.05 | 25 | 50 | 1 | 0.9672 | 0.0256 | 0 |
| 2 | 0.05 | 25 | 50 | 2 | 1 | 0.844 | 0 |
| 1 | 0.2 | 25 | 50 | 1 | 0.8496 | 0.0692 | 0 |
| 1 | 0.2 | 25 | 50 | 2 | NA | NA | NA |
| 2 | 0.2 | 25 | 50 | 1 | 0.742 | 0.0924 | 0.0036 |
| 2 | 0.2 | 25 | 50 | 2 | 0.9484 | 0.4216 | 0.004 |

*Additional Table 4: Power (1- Type 2 error) and type 1 error of trials with two interims. The first interim occurred 50% through the trial, and the second interim 75% through the trial. ICC = intra-class correlation.*

|  |  |  |  | Proportion (±1 Monte Carlo Standard Error) | | |
| --- | --- | --- | --- | --- | --- | --- |
| N interims | ICC | N clusters per arm | N participants per cluster | Type 1 error | 1 - Type 2 error | 1 - Type 2 error (scaled) |
| 2 | 0.05 | 15 | 10 | 0.03 (0.026, 0.033) | 0.695 (0.686, 0.704) | 0.76 (0.751, 0.769) |
| 2 | 0.2 | 15 | 10 | 0.035 (0.032, 0.039) | 0.466 (0.456, 0.476) | 0.514 (0.504, 0.524) |
| 2 | 0.05 | 25 | 10 | 0.035 (0.032, 0.039) | 0.87 (0.863, 0.876) | 0.897 (0.891, 0.903) |
| 2 | 0.2 | 25 | 10 | 0.028 (0.025, 0.032) | 0.641 (0.631, 0.65) | 0.712 (0.703, 0.721) |
| 2 | 0.05 | 15 | 25 | 0.027 (0.024, 0.03) | 0.875 (0.869, 0.882) | 0.911 (0.905, 0.917) |
| 2 | 0.2 | 15 | 25 | 0.03 (0.027, 0.033) | 0.542 (0.532, 0.552) | 0.628 (0.619, 0.638) |
| 2 | 0.05 | 25 | 25 | 0.034 (0.03, 0.038) | 0.97 (0.967, 0.974) | 0.976 (0.973, 0.979) |
| 2 | 0.2 | 25 | 25 | 0.029 (0.025, 0.032) | 0.723 (0.714, 0.732) | 0.776 (0.768, 0.785) |
| 2 | 0.05 | 15 | 50 | 0.032 (0.029, 0.036) | 0.936 (0.931, 0.941) | 0.958 (0.954, 0.962) |
| 2 | 0.2 | 15 | 50 | 0.039 (0.035, 0.043) | 0.579 (0.569, 0.589) | 0.615 (0.605, 0.625) |
| 2 | 0.05 | 25 | 50 | 0.022 (0.019, 0.025) | 0.987 (0.985, 0.989) | 0.995 (0.994, 0.997) |
| 2 | 0.2 | 25 | 50 | 0.029 (0.025, 0.032) | 0.749 (0.741, 0.758) | 0.806 (0.798, 0.814) |

*Additional Table 5: Proportion of trials that stopped for futility over trial properties. The first interim occurred 50% through the trial, and the second interim 75% through the trial. ICC = intra-class correlation.*

|  |  |  |  |  | Proportion | | |
| --- | --- | --- | --- | --- | --- | --- | --- |
| Scenario | N interims | ICC | N clusters per arm | N participants per cluster | Stopped interim 1 | Stopped interim 2 | Stopped total |
| Null | 2 | 0.05 | 15 | 10 | 0.0476 | 0.0304 | 0.078 |
| Null | 2 | 0.2 | 15 | 10 | 0.068 | 0.0372 | 0.1052 |
| Null | 2 | 0.05 | 25 | 10 | 0.05 | 0.0224 | 0.0724 |
| Null | 2 | 0.2 | 25 | 10 | 0.0632 | 0.0284 | 0.0916 |
| Null | 2 | 0.05 | 15 | 25 | 0.056 | 0.0328 | 0.0888 |
| Null | 2 | 0.2 | 15 | 25 | 0.07 | 0.0396 | 0.1096 |
| Null | 2 | 0.05 | 25 | 25 | 0.054 | 0.0284 | 0.0824 |
| Null | 2 | 0.2 | 25 | 25 | 0.06 | 0.0344 | 0.0944 |
| Null | 2 | 0.05 | 15 | 50 | 0.0556 | 0.0376 | 0.0932 |
| Null | 2 | 0.2 | 15 | 50 | 0.0684 | 0.032 | 0.1004 |
| Null | 2 | 0.05 | 25 | 50 | 0.0488 | 0.0328 | 0.0816 |
| Null | 2 | 0.2 | 25 | 50 | 0.0608 | 0.0328 | 0.0936 |
| Effect | 2 | 0.05 | 15 | 10 | 0 | 0 | 0 |
| Effect | 2 | 0.2 | 15 | 10 | 0 | 0 | 0 |
| Effect | 2 | 0.05 | 25 | 10 | 0 | 0 | 0 |
| Effect | 2 | 0.2 | 25 | 10 | 0 | 0 | 0 |
| Effect | 2 | 0.05 | 15 | 25 | 0 | 0 | 0 |
| Effect | 2 | 0.2 | 15 | 25 | 0 | 0 | 0 |
| Effect | 2 | 0.05 | 25 | 25 | 0 | 0 | 0 |
| Effect | 2 | 0.2 | 25 | 25 | 0 | 0 | 0 |
| Effect | 2 | 0.05 | 15 | 50 | 0 | 0 | 0 |
| Effect | 2 | 0.2 | 15 | 50 | 0 | 0 | 0 |
| Effect | 2 | 0.05 | 25 | 50 | 0 | 0 | 0 |
| Effect | 2 | 0.2 | 25 | 50 | 0 | 0 | 0 |

*Additional Table 6: Proportion of trials that dropped a treatment arm by number of interims and trial properties in the effect scenario. ICC = intra-class correlation. The first interim occurred 50% through the trial, and the second interim 75% through the trial.*

|  |  |  |  |  | Proportion | | |
| --- | --- | --- | --- | --- | --- | --- | --- |
| N interims | ICC | N clusters per arm | N participants per cluster | Interim | Arm 2 | Arm 3 | Arm 4 |
| 2 | 0.05 | 15 | 10 | 1 | 0.7904 | 0.0836 | 0.002 |
| 2 | 0.05 | 15 | 10 | 2 | 0.948 | 0.4384 | 0.0024 |
| 2 | 0.2 | 15 | 10 | 1 | 0.626 | 0.1208 | 0.0064 |
| 2 | 0.2 | 15 | 10 | 2 | 0.8308 | 0.3316 | 0.01 |
| 2 | 0.05 | 25 | 10 | 1 | 0.9232 | 0.0508 | 0 |
| 2 | 0.05 | 25 | 10 | 2 | 0.9944 | 0.6032 | 0.0004 |
| 2 | 0.2 | 25 | 10 | 1 | 0.776 | 0.092 | 0.0044 |
| 2 | 0.2 | 25 | 10 | 2 | 0.9388 | 0.3936 | 0.0072 |
| 2 | 0.05 | 15 | 25 | 1 | 0.9204 | 0.0428 | 0.0004 |
| 2 | 0.05 | 15 | 25 | 2 | 0.99 | 0.6152 | 0.0004 |
| 2 | 0.2 | 15 | 25 | 1 | 0.6884 | 0.1 | 0.0044 |
| 2 | 0.2 | 15 | 25 | 2 | 0.876 | 0.3484 | 0.0072 |
| 2 | 0.05 | 25 | 25 | 1 | 0.98 | 0.0188 | 0 |
| 2 | 0.05 | 25 | 25 | 2 | 0.9996 | 0.8084 | 0 |
| 2 | 0.2 | 25 | 25 | 1 | 0.85 | 0.0636 | 0.0008 |
| 2 | 0.2 | 25 | 25 | 2 | 0.9724 | 0.4592 | 0.0016 |
| 2 | 0.05 | 15 | 50 | 1 | 0.9596 | 0.026 | 0 |
| 2 | 0.05 | 15 | 50 | 2 | 0.9984 | 0.7056 | 0.0004 |
| 2 | 0.2 | 15 | 50 | 1 | 0.7136 | 0.094 | 0.0028 |
| 2 | 0.2 | 15 | 50 | 2 | 0.894 | 0.3532 | 0.0068 |
| 2 | 0.05 | 25 | 50 | 1 | 0.994 | 0.006 | 0 |
| 2 | 0.05 | 25 | 50 | 2 | 1 | 0.8816 | 0 |
| 2 | 0.2 | 25 | 50 | 1 | 0.8692 | 0.064 | 0.0008 |
| 2 | 0.2 | 25 | 50 | 2 | 0.978 | 0.4764 | 0.002 |

*Additional Table 7: Power (1-Type 2 error) of the fixed and adaptive designs with 0 (fixed), 1, and 2 interims over trial properties in an unclear optimal arm scenario. ICC = intra-class correlation.*

|  |  |  |  | Proportion (±1 Monte Carlo Standard Error) | | |
| --- | --- | --- | --- | --- | --- | --- |
| N interims | ICC | N clusters per arm | N participants per cluster | Type 1 error | 1 - Type 2 error | 1 - Type 2 error (scaled) |
| 0 | 0.05 | 15 | 10 | 0.041 (0.037, 0.045) | 0.411 (0.401, 0.421) | 0.436 (0.426, 0.446) |
| 1 | 0.05 | 15 | 10 | 0.045 (0.041, 0.049) | 0.474 (0.464, 0.484) | 0.493 (0.483, 0.503) |
| 2 | 0.05 | 15 | 10 | 0.03 (0.026, 0.033) | 0.402 (0.392, 0.412) | 0.488 (0.478, 0.498) |
| 0 | 0.2 | 15 | 10 | 0.052 (0.048, 0.057) | 0.279 (0.27, 0.288) | 0.27 (0.261, 0.279) |
| 1 | 0.2 | 15 | 10 | 0.068 (0.063, 0.073) | 0.338 (0.328, 0.347) | 0.286 (0.277, 0.295) |
| 2 | 0.2 | 15 | 10 | 0.031 (0.027, 0.034) | 0.256 (0.247, 0.264) | 0.298 (0.289, 0.308) |
| 0 | 0.05 | 25 | 10 | 0.051 (0.047, 0.056) | 0.597 (0.587, 0.607) | 0.592 (0.582, 0.601) |
| 1 | 0.05 | 25 | 10 | 0.045 (0.041, 0.049) | 0.638 (0.628, 0.647) | 0.651 (0.641, 0.66) |
| 2 | 0.05 | 25 | 10 | 0.029 (0.025, 0.032) | 0.566 (0.556, 0.576) | 0.632 (0.623, 0.642) |
| 0 | 0.2 | 25 | 10 | 0.064 (0.059, 0.069) | 0.436 (0.426, 0.446) | 0.401 (0.391, 0.411) |
| 1 | 0.2 | 25 | 10 | 0.046 (0.041, 0.05) | 0.458 (0.448, 0.468) | 0.477 (0.467, 0.487) |
| 2 | 0.2 | 25 | 10 | 0.034 (0.03, 0.038) | 0.373 (0.363, 0.382) | 0.447 (0.437, 0.457) |
| 0 | 0.05 | 15 | 25 | 0.06 (0.056, 0.065) | 0.628 (0.618, 0.637) | 0.603 (0.593, 0.613) |
| 1 | 0.05 | 15 | 25 | 0.05 (0.046, 0.054) | 0.689 (0.68, 0.698) | 0.689 (0.68, 0.698) |
| 2 | 0.05 | 15 | 25 | 0.028 (0.025, 0.031) | 0.628 (0.618, 0.638) | 0.716 (0.707, 0.725) |
| 0 | 0.2 | 15 | 25 | 0.062 (0.057, 0.066) | 0.348 (0.338, 0.357) | 0.318 (0.309, 0.328) |
| 1 | 0.2 | 15 | 25 | 0.048 (0.044, 0.052) | 0.4 (0.39, 0.409) | 0.403 (0.393, 0.413) |
| 2 | 0.2 | 15 | 25 | 0.032 (0.029, 0.036) | 0.302 (0.293, 0.312) | 0.384 (0.375, 0.394) |
| 0 | 0.05 | 25 | 25 | 0.05 (0.046, 0.055) | 0.779 (0.771, 0.787) | 0.779 (0.771, 0.787) |
| 1 | 0.05 | 25 | 25 | 0.049 (0.044, 0.053) | 0.851 (0.844, 0.858) | 0.852 (0.844, 0.859) |
| 2 | 0.05 | 25 | 25 | 0.032 (0.028, 0.035) | 0.808 (0.801, 0.816) | 0.855 (0.848, 0.862) |
| 0 | 0.2 | 25 | 25 | 0.052 (0.048, 0.057) | 0.518 (0.508, 0.528) | 0.511 (0.501, 0.521) |
| 1 | 0.2 | 25 | 25 | 0.053 (0.048, 0.057) | 0.565 (0.555, 0.575) | 0.556 (0.546, 0.566) |
| 2 | 0.2 | 25 | 25 | 0.024 (0.021, 0.027) | 0.484 (0.474, 0.494) | 0.582 (0.572, 0.592) |
| 0 | 0.05 | 15 | 50 | 0.054 (0.049, 0.059) | 0.74 (0.732, 0.749) | 0.73 (0.721, 0.739) |
| 1 | 0.05 | 15 | 50 | 0.05 (0.045, 0.054) | 0.802 (0.794, 0.81) | 0.805 (0.797, 0.813) |
| 2 | 0.05 | 15 | 50 | 0.025 (0.022, 0.028) | 0.755 (0.747, 0.764) | 0.814 (0.806, 0.822) |
| 0 | 0.2 | 15 | 50 | 0.056 (0.051, 0.061) | 0.386 (0.376, 0.395) | 0.368 (0.358, 0.377) |
| 1 | 0.2 | 15 | 50 | 0.056 (0.051, 0.06) | 0.441 (0.431, 0.451) | 0.426 (0.416, 0.436) |
| 2 | 0.2 | 15 | 50 | 0.029 (0.025, 0.032) | 0.363 (0.353, 0.372) | 0.438 (0.428, 0.448) |
| 0 | 0.05 | 25 | 50 | 0.056 (0.051, 0.061) | 0.878 (0.872, 0.885) | 0.867 (0.86, 0.874) |
| 1 | 0.05 | 25 | 50 | 0.045 (0.041, 0.049) | 0.927 (0.922, 0.932) | 0.931 (0.926, 0.936) |
| 2 | 0.05 | 25 | 50 | 0.03 (0.027, 0.034) | 0.892 (0.885, 0.898) | 0.936 (0.931, 0.941) |
| 0 | 0.2 | 25 | 50 | 0.055 (0.051, 0.06) | 0.546 (0.536, 0.556) | 0.525 (0.515, 0.535) |
| 1 | 0.2 | 25 | 50 | 0.053 (0.049, 0.058) | 0.605 (0.595, 0.615) | 0.602 (0.592, 0.611) |
| 2 | 0.2 | 25 | 50 | 0.022 (0.019, 0.025) | 0.517 (0.507, 0.527) | 0.634 (0.624, 0.644) |

*Additional Table 8: Proportion of trials that stopped for futility over trial properties in an unclear optimal arm scenario. ICC = intra-class correlation.*

|  |  |  |  | Proportion | | |
| --- | --- | --- | --- | --- | --- | --- |
| N interims | ICC | N clusters per arm | N participants per cluster | Stopped interim 1 | Stopped interim 2 | Stopped total |
| 1 | 0.05 | 15 | 10 | 0.0024 | NA | 0.0024 |
| 2 | 0.05 | 15 | 10 | 0.0052 | 0.0004 | 0.0056 |
| 1 | 0.2 | 15 | 10 | 0.0076 | NA | 0.0076 |
| 2 | 0.2 | 15 | 10 | 0.0116 | 0.0028 | 0.0144 |
| 1 | 0.05 | 25 | 10 | 0 | NA | 0 |
| 2 | 0.05 | 25 | 10 | 0.002 | 0 | 0.002 |
| 1 | 0.2 | 25 | 10 | 0.0028 | NA | 0.0028 |
| 2 | 0.2 | 25 | 10 | 0.0064 | 0.0008 | 0.0072 |
| 1 | 0.05 | 15 | 25 | 0.0004 | NA | 0.0004 |
| 2 | 0.05 | 15 | 25 | 0.0004 | 0 | 0.0004 |
| 1 | 0.2 | 15 | 25 | 0.0076 | NA | 0.0076 |
| 2 | 0.2 | 15 | 25 | 0.0112 | 0.0004 | 0.0116 |
| 1 | 0.05 | 25 | 25 | 0 | NA | 0 |
| 2 | 0.05 | 25 | 25 | 0.0004 | 0 | 0.0004 |
| 1 | 0.2 | 25 | 25 | 0.0012 | NA | 0.0012 |
| 2 | 0.2 | 25 | 25 | 0.0032 | 0 | 0.0032 |
| 1 | 0.05 | 15 | 50 | 0 | NA | 0 |
| 2 | 0.05 | 15 | 50 | 0 | 0 | 0 |
| 1 | 0.2 | 15 | 50 | 0.0056 | NA | 0.0056 |
| 2 | 0.2 | 15 | 50 | 0.0092 | 0.0008 | 0.01 |
| 1 | 0.05 | 25 | 50 | 0 | NA | 0 |
| 2 | 0.05 | 25 | 50 | 0 | 0 | 0 |
| 1 | 0.2 | 25 | 50 | 0.0004 | NA | 0.0004 |
| 2 | 0.2 | 25 | 50 | 0.0032 | 0 | 0.0032 |

*Additional Table 9: Proportion of trials that dropped a treatment arm by number of interims and trial properties in an unclear optimal arm scenario. ICC = intra-class correlation.*

|  |  |  |  |  | Proportion | | |
| --- | --- | --- | --- | --- | --- | --- | --- |
| Number of interims | IICC | N clusters per arm | N participants per cluster | Interim | Arm 2 | Arm 3 | Arm 4 |
| 1 | 0.05 | 15 | 10 | 1 | 0.6144 | 0.1092 | 0.0116 |
| 1 | 0.05 | 15 | 10 | 2 | NA | NA | NA |
| 2 | 0.05 | 15 | 10 | 1 | 0.5424 | 0.136 | 0.0184 |
| 2 | 0.05 | 15 | 10 | 2 | 0.8008 | 0.3164 | 0.0236 |
| 1 | 0.2 | 15 | 10 | 1 | 0.5464 | 0.1436 | 0.0304 |
| 1 | 0.2 | 15 | 10 | 2 | NA | NA | NA |
| 2 | 0.2 | 15 | 10 | 1 | 0.4988 | 0.1592 | 0.0464 |
| 2 | 0.2 | 15 | 10 | 2 | 0.7204 | 0.3184 | 0.062 |
| 1 | 0.05 | 25 | 10 | 1 | 0.7328 | 0.11 | 0.0028 |
| 1 | 0.05 | 25 | 10 | 2 | NA | NA | NA |
| 2 | 0.05 | 25 | 10 | 1 | 0.6332 | 0.12 | 0.0096 |
| 2 | 0.05 | 25 | 10 | 2 | 0.8956 | 0.3588 | 0.012 |
| 1 | 0.2 | 25 | 10 | 1 | 0.6352 | 0.1288 | 0.0128 |
| 1 | 0.2 | 25 | 10 | 2 | NA | NA | NA |
| 2 | 0.2 | 25 | 10 | 1 | 0.5624 | 0.1444 | 0.0276 |
| 2 | 0.2 | 25 | 10 | 2 | 0.7956 | 0.3208 | 0.036 |
| 1 | 0.05 | 15 | 25 | 1 | 0.7856 | 0.0724 | 0.0028 |
| 1 | 0.05 | 15 | 25 | 2 | NA | NA | NA |
| 2 | 0.05 | 15 | 25 | 1 | 0.6996 | 0.104 | 0.0048 |
| 2 | 0.05 | 15 | 25 | 2 | 0.926 | 0.3952 | 0.0064 |
| 1 | 0.2 | 15 | 25 | 1 | 0.602 | 0.122 | 0.0208 |
| 1 | 0.2 | 15 | 25 | 2 | NA | NA | NA |
| 2 | 0.2 | 15 | 25 | 1 | 0.5452 | 0.1452 | 0.0272 |
| 2 | 0.2 | 15 | 25 | 2 | 0.7652 | 0.3128 | 0.0368 |
| 1 | 0.05 | 25 | 25 | 1 | 0.9084 | 0.0524 | 0 |
| 1 | 0.05 | 25 | 25 | 2 | NA | NA | NA |
| 2 | 0.05 | 25 | 25 | 1 | 0.8036 | 0.0816 | 0.0024 |
| 2 | 0.05 | 25 | 25 | 2 | 0.9752 | 0.5056 | 0.0036 |
| 1 | 0.2 | 25 | 25 | 1 | 0.7228 | 0.0964 | 0.006 |
| 1 | 0.2 | 25 | 25 | 2 | NA | NA | NA |
| 2 | 0.2 | 25 | 25 | 1 | 0.6216 | 0.1244 | 0.0136 |
| 2 | 0.2 | 25 | 25 | 2 | 0.8596 | 0.338 | 0.0176 |
| 1 | 0.05 | 15 | 50 | 1 | 0.8848 | 0.0536 | 8e-04 |
| 1 | 0.05 | 15 | 50 | 2 | NA | NA | NA |
| 2 | 0.05 | 15 | 50 | 1 | 0.796 | 0.0812 | 0.004 |
| 2 | 0.05 | 15 | 50 | 2 | 0.9672 | 0.4712 | 0.0052 |
| 1 | 0.2 | 15 | 50 | 1 | 0.6376 | 0.1104 | 0.012 |
| 1 | 0.2 | 15 | 50 | 2 | NA | NA | NA |
| 2 | 0.2 | 15 | 50 | 1 | 0.5652 | 0.1392 | 0.0244 |
| 2 | 0.2 | 15 | 50 | 2 | 0.7956 | 0.3196 | 0.032 |
| 1 | 0.05 | 25 | 50 | 1 | 0.9608 | 0.0288 | 0 |
| 1 | 0.05 | 25 | 50 | 2 | NA | NA | NA |
| 2 | 0.05 | 25 | 50 | 1 | 0.908 | 0.048 | 0 |
| 2 | 0.05 | 25 | 50 | 2 | 0.9944 | 0.5972 | 8e-04 |
| 1 | 0.2 | 25 | 50 | 1 | 0.762 | 0.092 | 0.0032 |
| 1 | 0.2 | 25 | 50 | 2 | NA | NA | NA |
| 2 | 0.2 | 25 | 50 | 1 | 0.6596 | 0.11 | 0.012 |
| 2 | 0.2 | 25 | 50 | 2 | 0.8896 | 0.3256 | 0.0144 |
