# Supplementary material for: Adaptive designs for trials aiming to optimise implementation strategies and the effect of an additional interim analysis: a simulation study
Source: BMC Med Res Methodol. 2025 Nov 29;26:1. doi: 10.1186/s12874-025-02730-y (PMC12771792; doi:10.1186/s12874-025-02730-y)
Supplement: Supplementary file 3 — Supplementary Material 3. Additional File 3, Examples of adaptive trials [file 12874_2025_2730_MOESM3_ESM.docx]

**Additional File 3 – Examples of adaptive trials**

**Example 1**

Trial properties:

- Effect scenario – Arm One = 0.1, Arm Two = 0.2, Arm Three = 0.3, Arm Four = 0.4.
- ICC = 0.05
- N participants per cluster = 25
- N clusters = 15
- One interim analysis

***Table 1:*** *Number of clusters randomised to each arm and the crude proportion of favourable events by trial timing and arm in example 1.*

|  | N clusters randomised | | Crude proportion of favourable event | |
| --- | --- | --- | --- | --- |
| Arm | Start of trial | After Interim One | Interim One | End of trial |
| One | 7 | 8 | 0.08 | 0.08 |
| Two | 7 | 0 | 0.12 | *NA* |
| Three | 7 | 12 | 0.40 | 0.33 |
| Four | 7 | 12 | 0.47 | 0.42 |

At interim one, the crude proportion of participants that had the favourable event was 0.08 in Arm One, 0.12 in Arm Two, 0.40 in Arm Three, and 0.47 in Arm Four (Table 1). At interim one, the odds ratio of a favourable event was 1.47 for Arm Two, 7.38 for Arm Three, and 9.79 for Arm Four. Arm Two had a posterior probability of success < 0.05 and therefore was dropped.

***Table 2:*** *Trial decisions, posterior probabilities and effect estimates at interim analysis one and the end of trial in example 1.*

| *Timing* | Probability of success | | | Odds ratio^1^ (95% CrI^2^) | | |
| --- | --- | --- | --- | --- | --- | --- |
| Decision | Arm Two | Arm Three | Arm Four | Arm Two | Arm Three | Arm Four |
| *Interim One* | | | | | | |
| Drop Arm Two | 0 | 0.11 | 0.89 | 1.47 (0.72, 3.03) | 7.38 (4.00, 14.0) | 9.79 (5.32, 18.3) |
| *Final analysis* | | | | | | |
| Arm Four optimal | *NA* | 0.003 | 0.99 | *NA* | 5.53 (3.64, 8.64) | 8.12 (5.44, 12.3) |

^1^ Reference group = Arm One; ^2^ Credible interval.

After the interim analysis, Arm One received its pre-specified fixed 8 clusters. The remaining treatment arms (that weren’t dropped), Arms Three and Four, each received their pre-specified 8 clusters, plus an additional 4 clusters that would have been allocated to Arm Two. This means, at the end of the trial:

- Arm One had 15 clusters
- Arm Two had 7 clusters
- Arm Three had 19 clusters
- Arm Four had 19 clusters

At the final analysis the odds of a favourable event were 8.12 times higher in Arm Four compared to Arm One. Arm Four had a posterior probability of success > 0.85. Thus, it was concluded that Arm Four was the optimal arm.

**Example 2**

- Null scenario – Arm One = 0.1, Arm Two = 0.1, Arm Three = 0.1, Arm Four = 0.1.
- ICC = 0.05
- N participants per cluster = 25
- N clusters = 15
- One interim analysis

***Table 3:*** *Number of clusters randomised to each arm and the crude proportion of favourable events by trial timing and arm in example 2.*

|  | N clusters randomised | | Crude proportion of favourable event | |
| --- | --- | --- | --- | --- |
| Arm | Start of trial | After Interim One | Interim One | End of trial |
| One | 7 | 0 | 0.14 | *NA* |
| Two | 7 | 0 | 0.10 | *NA* |
| Three | 7 | 0 | 0.10 | *NA* |
| Four | 7 | 0 | 0.09 | *NA* |

At interim one, the crude proportion of participants that had the favourable outcome was 0.14 in Arm One, 0.10 in Arm Two, 0.10 in Arm Three, and 0.09 in Arm Four (Table 3). All treatment arms had a probability of success < 0.15, and thus the trial was stopped early for futility (Table 4).

***Table 4:*** *Trial decisions, posterior probabilities and effect estimates at interim analysis one and the end of trial in example 1.*

| *Timing* | Probability of success | | | Odds ratio^1^ (95% CrI^2^) | | |
| --- | --- | --- | --- | --- | --- | --- |
| Decision | Arm Two | Arm Three | Arm Four | Arm Two | Arm Three | Arm Four |
| *Interim One* | | | | | | |
| Stop early for futility | 0.08 | 0.14 | 0.04 | 0.66 (0.32, 1.36) | 0.71 (0.34, 1.45) | 0.57 (0.26, 1.25) |

^1^ Reference group = Arm One; ^2^ Credible interval.

Since the trial stopped early for futility, no additional clusters were randomised.
