# Supplementary material for: Adaptive designs for trials aiming to optimise implementation strategies and the effect of an additional interim analysis: a simulation study
Source: BMC Med Res Methodol. 2025 Nov 29;26:1. doi: 10.1186/s12874-025-02730-y (PMC12771792; doi:10.1186/s12874-025-02730-y)

Additional File 4: *Proportion of trials that dropped a treatment arm by number of interims and trial properties in the null scenario. ICC = intra-class correlation.*


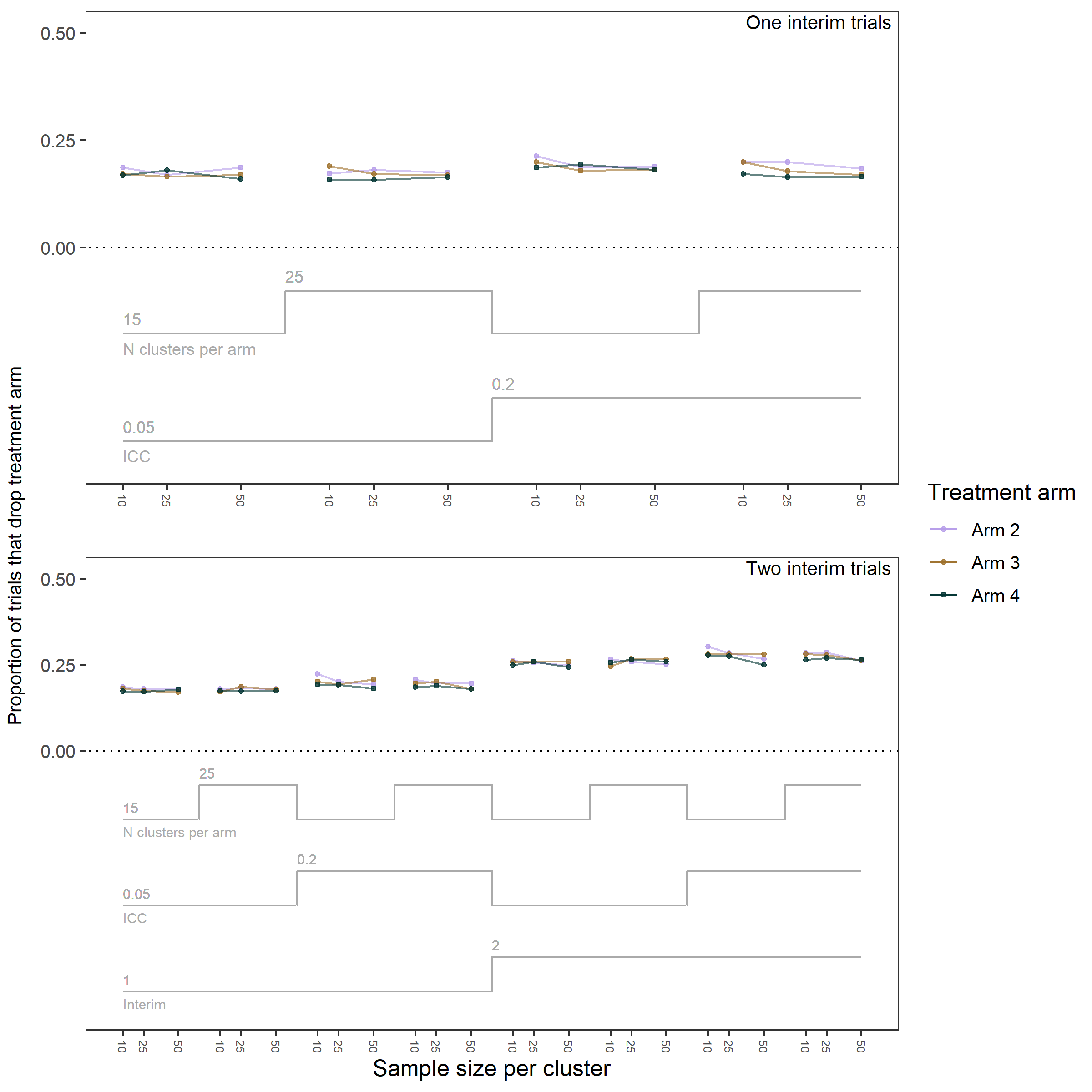

Supplement: Supplementary file 4 — Supplementary Material 4. Additional File 4, Proportion of trials that dropped a treatment arm by number of interims and trial properties in the null scenario. ICC = intra-class correlation [file 12874_2025_2730_MOESM4_ESM.docx]
